# TWEAK-Independent Fn14 Self-Association and NF-κB Activation Is Mediated by the C-Terminal Region of the Fn14 Cytoplasmic Domain

**DOI:** 10.1371/journal.pone.0065248

**Published:** 2013-06-04

**Authors:** Sharron A. N. Brown, Emily Cheng, Mark S. Williams, Jeffrey A. Winkles

**Affiliations:** 1 Department of Surgery, Center for Vascular and Inflammatory Diseases, University of Maryland School of Medicine, Baltimore, Maryland, United States of America; 2 Department of Microbiology and Immunology, Center for Vascular and Inflammatory Diseases, University of Maryland School of Medicine, Baltimore, Maryland, United States of America; University of Pittsburgh School of Medicine, United States of America

## Abstract

The tumor necrosis factor (TNF) superfamily member TNF-like weak inducer of apoptosis (TWEAK) is a pro-inflammatory and pro-angiogenic cytokine implicated in physiological tissue regeneration and wound repair. TWEAK binds to a 102-amino acid type I transmembrane cell surface receptor named fibroblast growth factor-inducible 14 (Fn14). TWEAK:Fn14 engagement activates several intracellular signaling cascades, including the NF-κB pathway, and sustained Fn14 signaling has been implicated in the pathogenesis of chronic inflammatory diseases and cancer. Although several groups are developing TWEAK- or Fn14-targeted agents for therapeutic use, much more basic science research is required before we fully understand the TWEAK/Fn14 signaling axis. For example, we and others have proposed that TWEAK-independent Fn14 signaling may occur in cells when Fn14 levels are highly elevated, but this idea has never been tested directly. In this report, we first demonstrate TWEAK-independent Fn14 signaling by showing that an Fn14 deletion mutant that is unable to bind TWEAK can activate the NF-κB pathway in transfected cells. We then show that ectopically-expressed, cell surface-localized Fn14 can self-associate into Fn14 dimers, and we show that Fn14 self-association is mediated by an 18-aa region within the Fn14 cytoplasmic domain. Endogenously-expressed Fn14 as well as ectopically-overexpressed Fn14 could also be detected in dimeric form when cell lysates were subjected to SDS-PAGE under non-reducing conditions. Additional experiments revealed that Fn14 dimerization occurs during cell lysis via formation of an intermolecular disulfide bond at cysteine residue 122. These findings provide insight into the Fn14 signaling mechanism and may aid current studies to develop therapeutic agents targeting this small cell surface receptor.

## Introduction

Fibroblast growth factor-inducible 14 (Fn14) was first described in 1999 as a growth factor-inducible, immediately-early gene predicted to encode a 129-aa type I transmembrane protein that would be cleaved intracellularly by signal peptidase into a mature 102-aa protein of unknown biological function [1], [2]. Shortly after these initial Fn14 studies were published, Wiley et al. [3] reported that the TNF superfamily member TWEAK could bind to Fn14 with low nanomolar affinity and, as predicted from this result, that Fn14 had several structural characteristics that supported its classification as a new member of the TNF receptor (TNFR) superfamily. TWEAK, a multifunctional cytokine that can induce either cell death, proliferation, survival, or differentiation, depending on the cellular context (reviewed in [4], [5]), is the only TNF superfamily member that can bind Fn14 [6]. TWEAK:Fn14 engagement has been shown to promote TNFR associated factor (TRAF) binding [7] and activation of a number of intracellular signal transduction cascades, including the ERK1/2 [8]–[10], PI3K/Akt [11], and NF-κB [8]–[10], [12]–[16] pathways. Studies using TWEAK- or Fn14-deficient mice have revealed that TWEAK/Fn14 signaling is not required for embryonic development or postnatal growth [17], [18] but may be critical for wound repair following acute tissue injury [15], [18], [19].

The TWEAK/Fn14 axis has been implicated in various human diseases. For example, recent work using several mouse models of human chronic inflammatory disease has indicated that TWEAK activity may exacerbate disease progression (reviewed in [4], [5]). Indeed, a Phase II clinical trial is in progress to test whether an anti-TWEAK monoclonal antibody may be a beneficial therapeutic agent for lupus nephritis patients (ClinicalTrials.gov Identifier NCT01499355). TWEAK and Fn14 may also be targets for cancer therapy (reviewed in [4], [20], [21], [22]). Of particular interest, Fn14 gene expression is elevated in over a dozen different solid tumor types compared with matched adjacent normal tissue or normal tissues from non-diseased donors [12], [23]–[26]. TWEAK/Fn14 signaling can have anti-tumorigenic effects (reviewed in [4], [22]); for example, TWEAK is a pro-apoptotic factor for some human cancer cell lines, and two companies have developed agonistic Fn14 antibodies that can kill cancer cells *in vitro* and inhibit xenograft tumor growth *in vivo* [23], [26]–[28]. TWEAK/Fn14 signaling can also have pro-tumorigenic effects (reviewed in [4], [22]) [11], [12], [29]; accordingly, in pre-clinical studies we have demonstrated that Fn14-targeted immunotoxins have anti-tumor activity [25], [30] and Hoffmann-LaRoche Inc. has initiated a Phase I clinical trial testing the effects of an anti-TWEAK monoclonal antibody in patients with advanced solid tumors (ClinicalTrials.gov Identifier NCT01383733).

A complete understanding of TWEAK/Fn14 signaling is critical for the optimal design of TWEAK/Fn14-directed therapeutic agents. One outstanding issue is whether Fn14 “activation” can occur not only in response to ligand binding (TWEAK-dependent Fn14 signaling) but also when Fn14 expression is elevated (TWEAK-independent Fn14 signaling). This latter signaling mechanism may be particularly important in cancers where Fn14 levels are high but TWEAK levels are low (e.g., in glioblastomas [31] and melanomas (unpublished data)). The main evidence in support of TWEAK-independent signaling is that experimental manipulation of Fn14 expression levels in cancer cells cultured *in vitro* can regulate signal transduction pathways [32] and cellular properties; for example, cell survival, migration and invasion [12], [24], [32]–[36]. However, these results do not conclusively demonstrate TWEAK-independent Fn14 signaling for two main reasons. First, the cells were grown in culture medium containing serum, a potential source of TWEAK [37], [38], and second, the cells themselves could be expressing TWEAK, and in particular, they could be releasing the soluble TWEAK isoform into the medium. In this report, we directly demonstrate TWEAK-independent Fn14 signaling by showing that an Fn14 deletion mutant encoded by an Fn14 splice variant mRNA is unable to bind TWEAK but can still activate the NF-κB pathway in transfected cells. We then show that ectopically-expressed, cell surface-localized Fn14 can self-associate into Fn14 dimers, and this dimerization is mediated by a region within the Fn14 cytoplasmic tail. Finally, we present additional evidence that Fn14 monomers are self-associated in cells by demonstrating that Fn14 dimers can be detected when cell lysates are examined under non-reducing gel conditions. These dimers form when cells are lysed and are due to the formation of a single intermolecular disulfide bond at Fn14 cysteine residue 122 in the cytoplasmic tail.

## Materials and Methods

### Cell Culture

HEK293 cells (ATCC) were grown in EMEM supplemented with 10% FBS, 1 mM sodium pyruvate, and 1X non-essential amino acids. HEK293/NF-κB-luc cells (Panomics) were grown in this same medium supplemented with 100 µg/ml hygromycin (CellGro). HEK293/NF-κB-luc cells engineered to stably overexpress HA epitope-tagged Fn14 [16] were grown in this same medium supplemented with 100 µg/ml hygromycin and 4 µg/ml blasticidin (Sigma). Human A172 glioma cells (ATCC), MDA-MB-231 breast cancer cells (ATCC) and A375 melanoma cells (from ATCC; provided by Dr. David Weber, University of Maryland School of Medicine) were grown in in DMEM supplemented with 10% FBS. Human HCC827 non-small cell lung cancer cells (ATCC) were grown in RPMI supplemented with 5% FBS. Human U87-luc glioma cells (provided by Dr. Andrew Kung, Dana Farber Cancer Institute) [39] were grown in DMEM supplemented with 10% FBS and 0.5 mg/ml G418 (Cellgro).

### RT-PCR and Construction of Human Fn14-myc Expression Plasmids

The expression plasmids encoding full-length, wild-type human Fn14 or an Fn14 mutant missing 35 amino acid residues in the extracellular domain (the ΔEC mutant) with N-terminal myc epitope tags were constructed as follows. First, RNA was isolated from human U87-luc and MDA-MB-231 cells using the RNeasy kit (Qiagen) and cDNA synthesized using the AccuScript RT-PCR system (Stratagene) according to the manufacturer’s instructions. Second, PCR was performed in the presence of 5% DMSO using a sense Fn14 primer targeting exon 1 nucleotides 150–176, an antisense primer targeting exon 4 nucleotides 30–56, and Vent Polymerase (New England Biolabs). Reaction products were separated by agarose gel electrophoresis and detected by ethidium bromide staining. Two DNA products were detected and the U87-luc products were isolated and ligated into pCMVScript (Stratagene). These plasmids were used as the template to add an optimal Kozak sequence (GCCGCCACC) prior to the ATG codon and to insert DNA encoding the myc epitope peptide (E-Q-K-L-I-S-E-E-D-L) at base 81, immediately 3′ to the DNA encoding the Fn14 signal peptide, by the PCR overlap extension method with Vent Polymerase. The final pCMVScript/Fn14-FL and pCMVScript/Fn14-ΔEC plasmids were then digested with *Not1/Xho1* and the released DNA fragments ligated into *Not1/Xho1*-digested pcDNA6 plasmid (Invitrogen). The expression plasmid encoding an Fn14 mutant missing the C-terminal 18 amino acids (the ΔCT mutant) was constructed using pCMVScript/Fn14-FL as a template and primers bracketing Fn14 amino acids 30 to 111. The sense primer contained an *Asc1* site and the antisense primer contained a stop codon and an *Xho1* site. The PCR product was cloned into pSecTag2A/Hygro (Invitrogen) as an *Asc1/Xho1* fragment to add the Ig-κ chain signal peptide in frame and then further cloned into *Not1/Xho1*-digested pcDNA6. The expression plasmids encoding Fn14 mutants with C104S or C122S amino acid substitutions were generated using overlapping PCR methodology with pCMVScript/Fn14-FL as the template and the appropriate primers. The PCR products were isolated, cloned into pCMVScript, and then subcloned as *Not1* fragments into pcDNA6. All expression constructs were subjected to DNA sequence analysis using appropriate primers and an Applied Biosystems automated sequencer.

### Plasmid DNA Transfections

Cells were transiently transfected using 10 µg plasmid DNA per 10-cm plate and the X-tremeGENE HP transfection reagent according to manufacturer’s instructions (Roche). Cells were generally harvested 24 hr post-transfection for subsequent analysis.

### Western Blot Analysis

Cells were lysed in HNTG buffer and protein concentrations determined as previously described [16]. In some experiments the cell lysis buffer was supplemented with 10 mM iodoacetamide (Sigma). Equal amounts of protein were subjected to SDS-PAGE under either reducing (DTT in loading buffer, sample is heated) or non-reducing (no DTT in loading buffer, sample is not heated) conditions and Western blot analysis conducted as described [16] except that proteins were transferred to PVDF, not nitrocellulose, membranes. The following primary antibodies were used: (a) anti-Fn14 polyclonal antibody (pAb) #3600 [1], (b) anti-Fn14 cytoplasmic tail pAb (Cell Signaling Technology), (c) anti-myc epitope monoclonal antibody (mAb) clone 9E10 (provided by Dr. Dudley Strickland, University of Maryland School of Medicine), (d) anti-HA epitope pAb (Clontech), (e) anti-tubulin mAb (Millipore), and (f) anti-GAPDH mAb (Cell Signaling Technology). Immunoreactive proteins were detected using Enhanced Chemiluminescence Plus (Amersham).

### Ligand Blot Analysis

HEK293 cells were transiently transfected with the pcDNA6 vector, the pcDNA6/Fn14-FL plasmid, or the pcDNA6/Fn14-ΔEC plasmid and harvested 24 hr post-transfection. Cells were lysed and equal amounts of protein were subjected to SDS-PAGE under non-reducing conditions as described above. The membrane was blocked and then sequentially incubated with 100 ng/ml recombinant human Fc-TWEAK fusion protein (provided by Dr. Pascal Schneider, University of Lausanne) and HRP-conjugated goat anti-human IgG/Fc secondary antibody (Millipore). The membrane was washed and immunoreactive Fc-TWEAK detected using Enhanced Chemiluminescence Plus.

### FACS Analysis

HEK293 cells were transiently transfected with the pcDNA6 vector, the pcDNA6/Fn14-FL plasmid, the pcDNA6/Fn14-ΔEC plasmid, or the pcDNA6/Fn14-ΔCT plasmid and harvested 24 hr post-transfection. Flow cytometry was conducted using FITC-labeled anti-myc epitope mAb (Miltenyi Biotec) as described previously [16].

### NF-κB Pathway Luciferase Reporter Assays

HEK293/NF-κB-luc cells were plated in normal growth medium in 6-well plates. The pcDNA6 vector, the pcDNA6/Fn14-FL plasmid, or the pcDNA6/Fn14-ΔEC plasmid were transfected in triplicate (1.5 µg/well) using X-tremeGENE HP as described above and harvested 24 hr later. Cells were lysed in Tropix Lysis Solution (Applied Biosystems). Aliquots of each cell lysate were combined with Luc-Screen System reagent (Applied Biosystems) and analysed in duplicate using an ML2250 microtiter plate luminometer (Dynatech Laboratories).

### Cell Surface Protein Cross-linking Experiments

HEK293/NF-κB-luc/Fn14-HA cells were transiently transfected with the pcDNA6 vector, the pcDNA6/Fn14-FL plasmid, the pcDNA6/Fn14-ΔEC plasmid, or the pcDNA6/Fn14-ΔCT plasmid and 24 hr later they were washed in PBS and then treated with 1 mM BS3 (Pierce) in PBS for 20 min at room temperature. The cross-linking reaction was quenched with 50 mM Tris-HCl (pH 7.4) for 15 min at room temperature and then the cells were harvested by scraping. Cells were lysed in HNTG buffer, and protein concentrations were determined using the BCA protein assay. Samples were subjected to SDS-PAGE under reducing conditions and Western blot analysis was performed.

### Co-immunoprecipitation Analysis

HEK293/NF-κB-luc/Fn14-HA cells were transiently transfected with the pcDNA6 vector, the pcDNA6/Fn14-FL plasmid, the pcDNA6/Fn14-ΔEC plasmid, or the pcDNA6/Fn14-ΔCT plasmid. Cells were harvested 24 hr post-transfection, lysed in HNTG buffer, and protein concentrations were determined using the BCA protein assay. An aliquot of each lysate was used for Western blot analysis. Co-immunoprecipitations were conducted using equal amounts of protein and the ProFound c-Myc Tag IP/Co-IP kit according to manufacturer’s instructions (Thermo Scientific). Samples were subjected to SDS-PAGE under reducing conditions and Western blot analysis was performed.

## Results and Discussion

### Identification of an Fn14 Splice Variant mRNA Predicted to Encode a Short Fn14 Isoform

**Figure 1:**
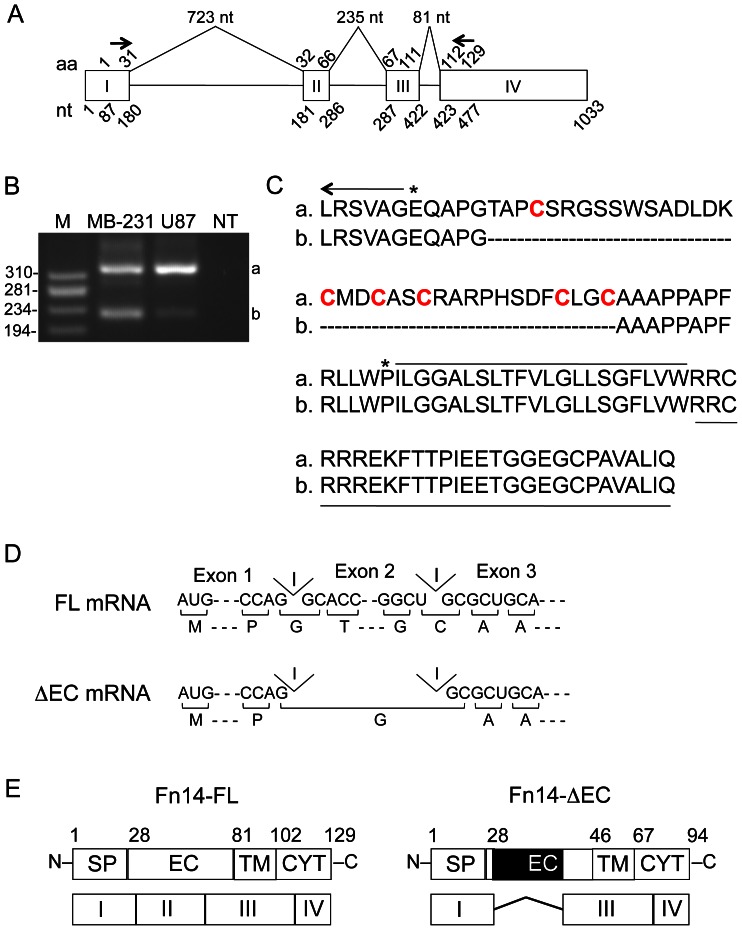
Cloning of a human Fn14 mRNA predicted to encode an Fn14 protein missing most of the extracellular domain. (**A**) Schematic representation of human Fn14 gene organization (via UCSC Genome Browser). The four Fn14 exons are numbered and boxed and the intron sizes are indicated in nucleotides (nt) at the top. The Fn14 amino acid (aa) numbers (1–129) and mature mRNA nucleotide (nt) numbers (1–1033) are provided above or below each exon, respectively. The positions of the two oligonucleotide primers used for RT-PCR analysis are indicated with arrows. (**B**) RNA was isolated from MDA-MB-231 and U87 cells and RT-PCR was performed using an exon 1 sense primer and an exon 4 antisense primer. PCR was also performed with this primer pair in the absence of cDNA (NT, no template). Amplification products were separated by agarose gel electrophoresis and visualized by ethidium bromide staining. The positions of DNA size markers (M) are shown on the left (in base pairs). The two PCR products that were isolated and sequenced are indicated on the right as a and b. (**C**) Predicted amino acid sequence of PCR amplification products a and b. The last six amino acids of the signal peptide are indicated with an arrow, the Fn14 extracellular domain is bracketed with asterisks and the six cysteine residues found in this domain are in red. The Fn14 transmembrane domain is overlined and the cytoplasmic tail is underlined. (**D**) The Fn14 mRNA translation initiation codon and selected codons surrounding Fn14 introns 1 and 2 are shown for the Fn14 full-length (Fn14-FL) and Fn14 extracellular domain deletion (Fn14-ΔEC) mRNAs. The predicted Fn14-FL and Fn14-ΔEC amino acid sequence is shown below the nucleotide sequence. Abbreviation: I, intron. (**E**) Schematic representation of the Fn14-FL and Fn14-ΔEC proteins showing structural domains in relation to their exon coding regions. Amino acid numbers corresponding to the beginning of each protein domain and the C-terminal amino acid are indicated at the top of each diagram. The region of the extracellular domain that is missing in Fn14-ΔEC is shown in black. Abbreviations: SP, signal peptide; EC, extracellular domain; TM, transmembrane domain; CYT, cytoplasmic tail.

The original goal of this study was to determine whether Fn14 protein-positive cancer cells expressed more than one Fn14 mRNA species. To test this, we performed RT-PCR analysis using RNA isolated from human MDA-MB-231 breast cancer cells and U87 glioma cells and exon 1- or exon 4-targeted oligonucleotide primers (Fig. 1A). Two major amplification products of 310- and 205-bp were detected (Fig. 1B). The U87 cell mRNA-derived DNA products were isolated, cloned and sequenced. The product A nucleotide sequence was 100% identical to the corresponding region of the human Fn14 cDNA sequence that we previously reported (GenBank Accession Number AF191148) [2]. This cDNA encodes full-length (129-aa) Fn14 (referred to here as Fn14-FL). The product B nucleotide sequence was 100% identical to the corresponding region of an unpublished human Fn14 cDNA sequence that was deposited in GenBank by S. Tanaka in 1999 (GenBank Accession Number AB035481). In comparison to product A, the product B sequence has a 105-bp deletion, and is predicted to encode a shorter Fn14 isoform missing an internal 35-aa region within the 53-aa extracellular domain (amino acids 33–67; we refer to this protein here as Fn14 extracellular domain deletion (Fn14-ΔEC) (Fig. 1C). Analysis of the human Fn14 exon sequences revealed that exon 2 is 105-bp in length and encodes these same 35 amino acids, suggesting that this exon is a cassette-type alternative exon (reviewed in [40]). Indeed, further analysis of the Fn14 gene exon/intron junction sequences is consistent with the proposal that alternative splicing of the Fn14 primary transcript via exon 2 skipping would generate the Fn14-ΔEC mRNA and protein represented by product B (Fig. 1D). The structural domains of the Fn14-FL and Fn14-ΔEC proteins are shown in reference to the Fn14 coding exons in Fig. 1E. We have been unable to detect an Fn14 protein species in MDA-MB-231 or U87 cells that we can conclusively identify as the Fn14-ΔEC protein by Western blotting using an antibody recognizing the Fn14 cytoplasmic tail (data not shown). Thus, this smaller Fn14 isoform may either not be expressed by these cells at all, or alternatively, it could be expressed at relatively low levels and not detected by the antibody we utilized.

### The Fn14-ΔEC Protein Cannot Bind TWEAK

**Figure 2:**
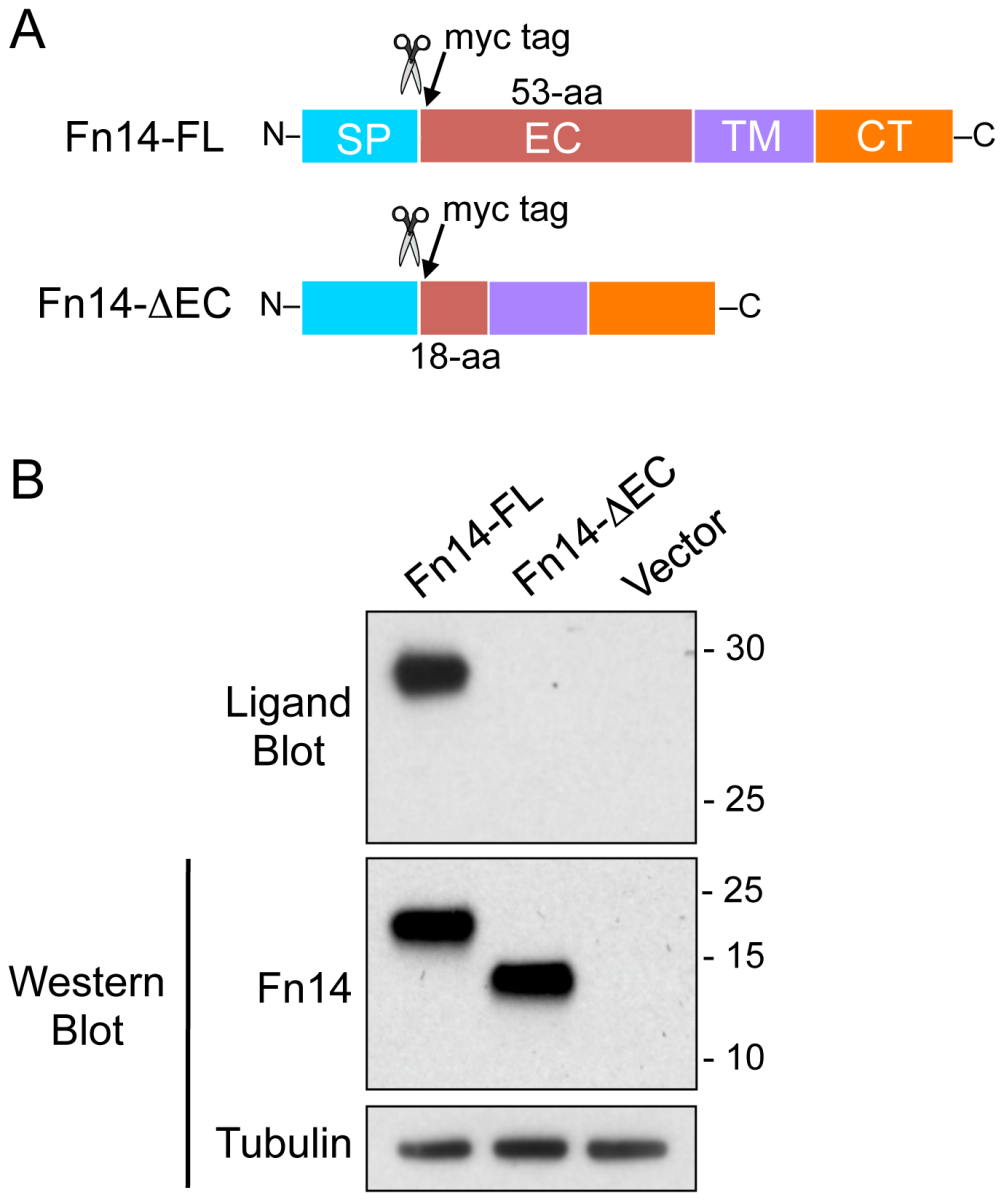
TWEAK cannot bind to the Fn14-ΔEC protein. (**A**) Schematic representation of the expression constructs encoding the Fn14-FL or Fn14-ΔEC protein. The Fn14 signal peptide (SP) region, extracellular (EC) domain, transmembrane (TM) domain, and cytoplasmic tail (CT) are indicated, the size of each EC domain is provided, and the positions of the signal peptide cleavage site (scissors) and the myc epitope tag are shown for both constructs. (**B**) HEK293 cells were transfected with vector or the indicated Fn14-myc expression plasmids. Cells were harvested 24 hr later, lysed, and equal amounts of protein were subjected to ligand blot analysis and Western blot analysis using either the anti-Fn14 #3600 antibody or an anti-tubulin antibody. The positions of molecular size markers are shown on the right (in kDa).

The Fn14-ΔEC isoform should not be able to bind TWEAK since it is missing most of the extracellular domain, and in particular, it does not have the cysteine-rich region within this domain that has been previously shown to contain the three disulfide bonds critical for proper Fn14 folding and TWEAK:Fn14 interaction [41]–[43]. We confirmed that TWEAK could not bind to Fn14-ΔEC using a ligand blot assay. Expression plasmids encoding either myc epitope-tagged Fn14-FL or Fn14-ΔEC (Fig. 2A), as well as the vector itself, were transfected into HEK293 cells. Cell lysates were prepared and protein was subjected to SDS-PAGE under non-reducing conditions (for ligand blot analysis) or reducing conditions (for Western blot analysis). TWEAK interaction with the Fn14-FL protein, but not the Fn14-ΔEC protein, was detected using the ligand blot assay, although Western blot analysis indicated that both proteins were expressed to a similar degree in the transfected cells (Fig. 2B).

### Fn14-ΔEC Overexpression in Transfected HEK293 Cells can Activate the NF-κB Signaling Pathway

**Figure 3:**
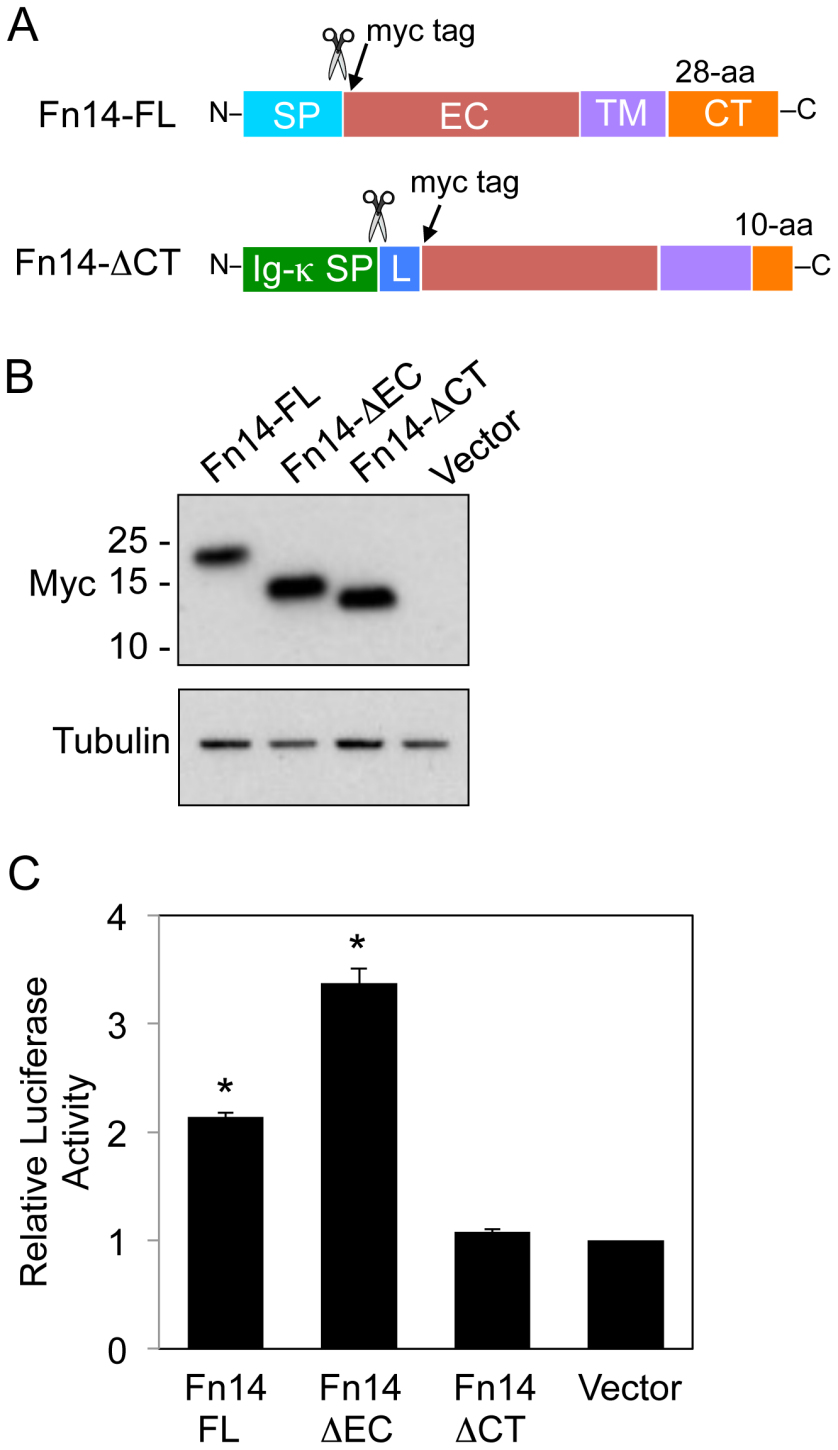
Transient Fn14-FL or Fn14-ΔEC overexpression, but not Fn14-ΔCT overexpression, can activate the NF-κB signaling pathway. (**A**) Schematic representation of the expression constructs encoding the Fn14-FL or Fn14-ΔCT protein. The Fn14-FL construct is the same as shown in Fig. 2A but here the size of the cytoplasmic tail (CT) is indicated. The Fn14-ΔCT construct contains an Ig-κ chain signal peptide instead of the Fn14 signal peptide and an extra stretch of 9-aa residues that was introduced during the cloning procedure (denoted here as L for linker). The positions of the signal peptide cleavage site (scissors) and the myc epitope tag are shown for both constructs. (**B**) HEK293 cells were transfected with vector or the indicated Fn14-myc expression plasmids and grown for 24 hr. Cells were harvested, lysed, and equal amounts of protein were used for Western blot analysis using either an anti-myc or anti-tubulin antibody. The positions of molecular size markers are shown on the left (in kDa). (**C**) HEK293/NF-κB-luc cells were transfected in 6 well dishes with the indicated plasmids and grown for 24 hr. The cells were harvested, lysed, and NF-κB-luc reporter expression was assayed using a luminometer. Luciferase activity was normalized to the vector-transfected cells. The values shown are mean +/− SEM of triplicate wells from one representative experiment of three independent experiments. *P<0.01 versus vector using Student’s t test.

There is substantial experimental evidence supporting the proposal that when cellular Fn14 levels are high, Fn14 can activate downstream signaling events without ligand engagement (a process referred to as TWEAK-independent Fn14 signaling (reviewed in [4]). However, the studies performed to date have not ruled out the possibility that the Fn14-triggered cellular effects observed, for example, NF-κB pathway activation [32], [44], [45], could be mediated by a low level of soluble TWEAK in the cell culture medium (either present in FBS [37], [38] or secreted by the cells under investigation). The cloning of Fn14-ΔEC afforded us the opportunity to directly test whether ectopic Fn14 overexpression promoted TWEAK-independent Fn14 signaling. As our readout for Fn14 signaling we selected NF-κB pathway activation, since TWEAK:Fn14 engagement in diverse cell types has been shown to stimulate this intracellular pathway (reviewed in [4]). HEK293/NF-κB-luc reporter cells were transiently transfected with vector or expression plasmids encoding either Fn14-FL (our positive control), Fn14-ΔEC, or Fn14-ΔCT, an Fn14 mutant that is missing the C-terminal 18-aa residues of the 28-aa Fn14 cytoplasmic tail (Fig. 3A) and therefore cannot bind TRAFs and activate signaling pathways (our negative control) [44]. All Fn14 proteins had an N-terminal Myc epitope tag. Cells were harvested 24 h later and processed for Western blot analysis and luciferase reporter assays. The three Fn14 proteins were expressed at similar levels (Fig. 3B). We noted previously that Fn14 does not migrate at its predicted molecular mass when analyzed by SDS-PAGE under reducing conditions [1] and this is again illustrated on this particular blot. For example, the predicted molecular mass (MM) of the mature, 112-aa myc-tagged Fn14-FL protein is 12.1-kDa, but its apparent MM on this gel is ∼23-kDa. Also, the 101-aa Fn14-ΔCT mutant (11.0-kDa predicted MM) is running at a lower apparent MM than the much smaller, 77-aa Fn14-ΔEC mutant (8.4-kDa predicted MM). The basis for these findings is not known but protein amino acid composition and isoelectric point are two interrelated factors that can influence the mobility of proteins on SDS polyacrylamide gels. For small proteins like Fn14, the effects of these factors on electrophoretic mobility are even more apparent. One possible explanation for the anomalous migration of the three Fn14 species relative to one another is that the Fn14-FL and Fn14-ΔEC proteins are acidic (predicted pIs of 5.8 and 5.3, respectively) while the Fn14-ΔCT mutant is basic (predicted pI of 8.8).

Transient overexpression of the Fn14-FL, Fn14-ΔEC, or Fn14-ΔCT proteins resulted in a 2.14-, 3.38- and 1.08-fold induction of the NF-κB pathway reporter gene, respectively (Fig. 3C). This finding demonstrates that TWEAK-independent Fn14 signaling can occur when cellular Fn14 levels are elevated. Although we used an ectopic overexpression approach to detect this signaling mechanism, it may also occur when Fn14 is naturally highly expressed, since we have reported previously that endogenous Fn14 down-regulation in cancer cells via RNA interference can alter cellular processes such as cell migration [24] and invasion [12], [24], [32].

### Fn14 Overexpression in Transfected HEK293 Cells Promotes Fn14 Self-association, which is Mediated by the C-terminal 18-aa Segment of the Fn14 Cytoplasmic Tail

**Figure 4:**
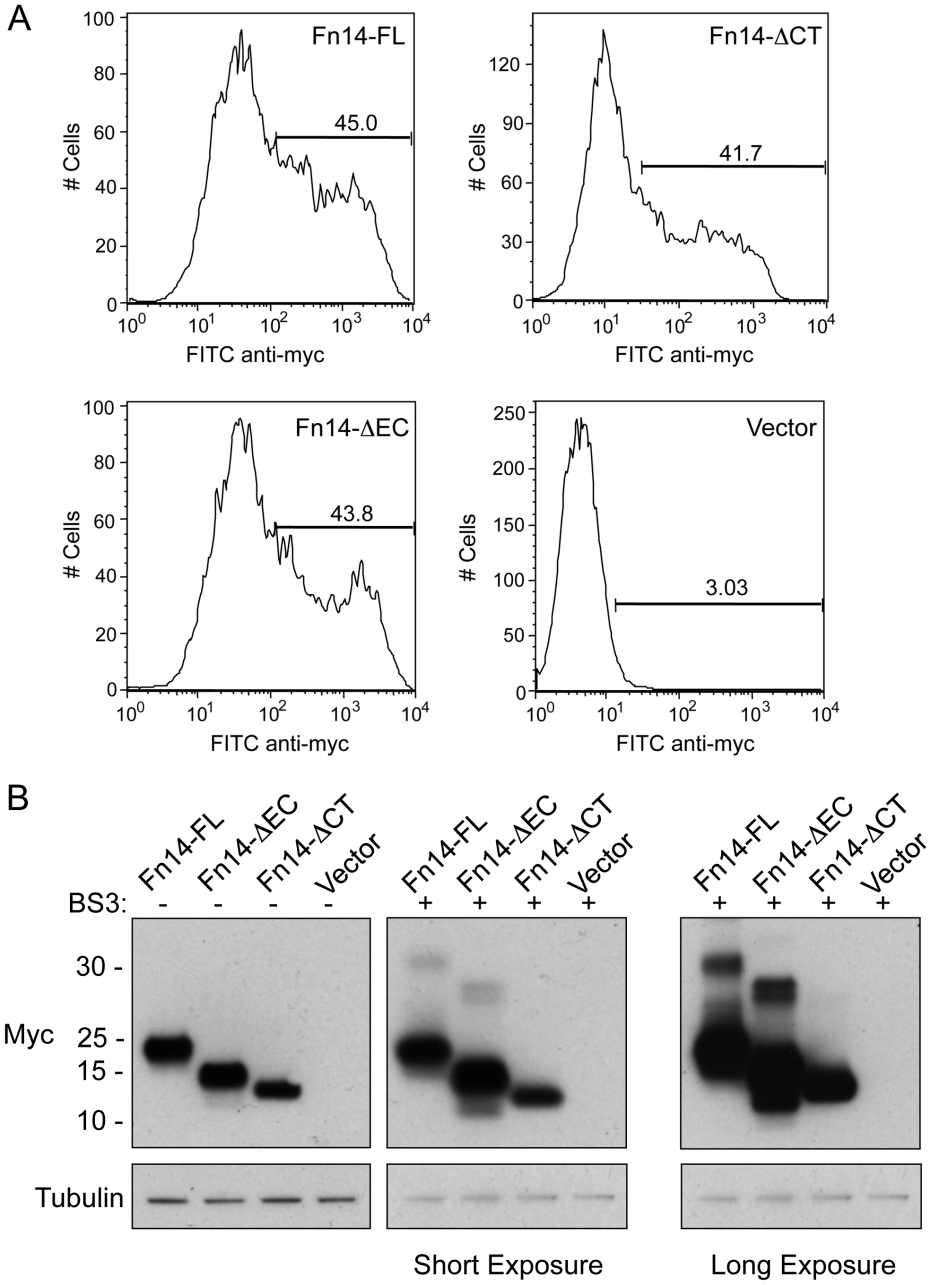
Transient Fn14-FL or Fn14-ΔEC overexpression, but not Fn14-ΔCT overexpression, promotes Fn14 self-association on the cell surface. (**A**) HEK293 cells were transfected with vector or each of the Fn14-myc expression plasmids. At 24 hr post-transfection the cells were stained with FITC-labeled anti-myc antibody and analyzed by flow cytometry. The markers indicate the percentage of myc-positive cells. The values shown are from one representative experiment of two independent experiments. (**B**) HEK293/NF-κB-luc/Fn14-HA cells were transfected with vector or the indicated Fn14-myc expression plasmids and 24 hr later the cells were either left untreated or treated with the cross-linking reagent BS3 as indicated. Cells were harvested, lysed, and equal amounts of protein were used for Western blot analysis using either an anti-myc or anti-tubulin antibody. The positions of molecular size markers are shown on the left (in kDa). Two different x-ray film exposures are shown for the BS3-treated samples.

The classic model of TNF:TNFR family signaling is that the binding of homotrimeric ligands promotes receptor trimerization which in turn leads to recruitment of signaling complexes to the cytoplasmic domains of the receptors (reviewed in [46]). However, it should be noted that the TNFR family member DR5 forms dimers, trimers and large multimers after ligand engagement [47]. Also, some TNFR family members (e.g., TNFR1 and CD40 [48], [49]) contain a pre-ligand assembly domain (PLAD) in their extracellular region that mediates non-covalent receptor association in the absence of ligand binding (reviewed in [50]). In the case of TNFR1, there is crystallographic evidence for receptor dimerization prior to ligand engagement [51]. The Fn14 extracellular region, only 53-aa in length, does not contain a PLAD domain, and there have been no studies reporting the structural organization of Fn14 either prior to ligand engagement, after ligand engagement, or when overexpressed in transfected cells. We previously proposed that the most likely explanation for TWEAK-independent Fn14 signaling is that when Fn14 is expressed at high levels in cells it will spontaneously self-associate, and this “receptor clustering” would promote TRAF association, downstream signaling and cellular responses (reviewed in [4]). To test if high Fn14 levels induce Fn14 self-association, our approach was to transfect cells and then cross-link all cell surface proteins using BS3, a membrane-impermeable, amine-to-amine crosslinking agent. Prior to conducting these experiments, we first determined if the Fn14-FL, Fn14-ΔEC, and Fn14-ΔCT proteins were all present on the HEK293 cell surface following transient transfection by performing FACS analysis with an anti-myc epitope tag antibody. We found that each protein was expressed on the cell surface to a similar degree (Fig. 4A). For the cross-linking experiments, cells were transiently transfected with vector or the Fn14-FL, Fn14-ΔEC, or Fn14-ΔCT expression plasmids and then either left untreated or treated with BS3. The cross-linking reaction was quenched and the cells were lysed and processed for Western blot analysis. We were able to detect the presence of cross-linked Fn14-FL and Fn14-ΔEC species at the approximate molecular weight of an Fn14 dimer in response to BS3 addition (Fig. 4B). A relatively small amount of each Fn14 protein was detected as cross-linked dimer. This indicates that only a small percentage of the Fn14 is self-associated or, alternatively, that the crosslinking reaction is inefficient (the only available primary amines in the Fn14-myc-overexpressing cells include the myc tag N-terminus and the side chain of lysine residue 3 (myc epitope tag) and lysine residue 31 (Fn14 extracellular domain)). In contrast to our findings regarding the Fn14-FL and Fn14-ΔEC proteins, ectopic overexpression of the Fn14-ΔCT protein and cross-linker addition did not generate a high molecular weight cross-linked Fn14 species. If we assume that Fn14-triggered NF-κB activation requires self-association followed by TRAF binding, then the inability of overexpressed Fn14-ΔCT protein to signal (Fig. 3C) may reflect ineffective self-association, not simply the absence of a TRAF binding site.

Taken together, these results indicate that (i) ectopic Fn14 overexpression can promote Fn14 self-association, (ii) Fn14 self-association is not ligand-dependent, and (iii) Fn14 self-association requires the most C-terminal 18-aa segment of the Fn14 cytoplasmic tail. Previous co-immunoprecipitation studies have revealed that ectopic overexpression of the TNFR superfamily members CD30 [52] and RANK [53] also results in receptor self-association. In the case of RANK, the self-association domain was mapped by deletion and site-specific mutagenesis to a 6-aa region in the cytoplasmic tail [53].

### The Fn14-ΔEC and Fn14-ΔCT Proteins can Both Associate with the Fn14-FL Protein

**Figure 5:**
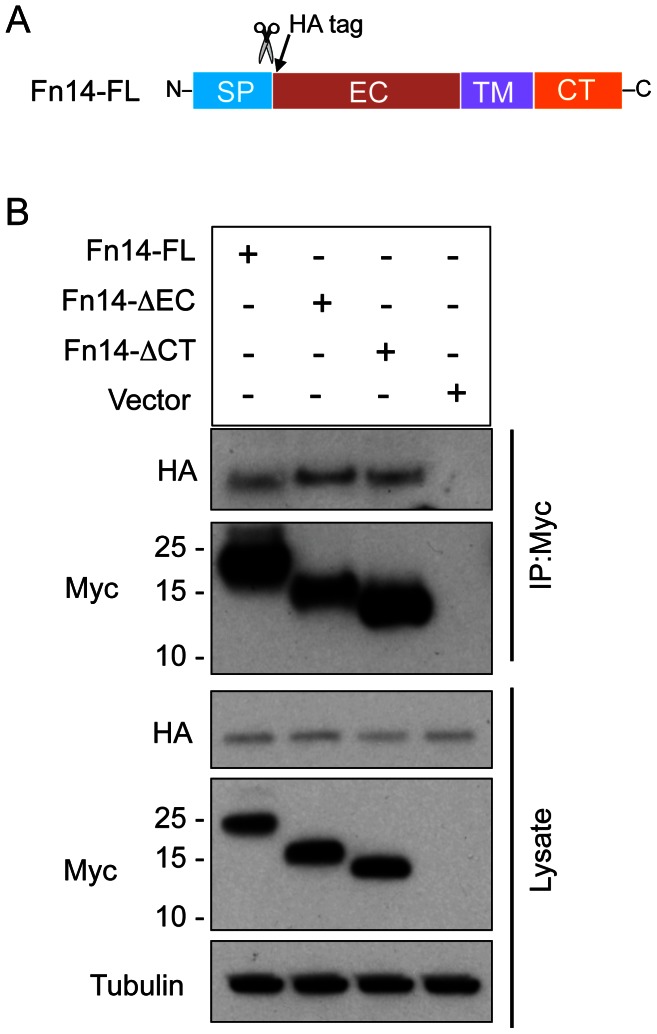
The Fn14-FL, Fn14-ΔEC, and Fn14-ΔCT proteins can all associate with the Fn14-FL protein. (**A**) Schematic representation of the expression construct encoding the HA-tagged Fn14-FL protein. The Fn14 signal peptide (SP) region, extracellular (EC) domain, transmembrane (TM) domain, and cytoplasmic tail (CT) are indicated and the positions of the signal peptide cleavage site (scissors) and the HA epitope tag are shown. (**B**) HEK293/NF-κB-luc/Fn14-HA cells were transfected with vector or the indicated Fn14-myc expression plasmids and 24 hr later the cells were harvested, lysed, and equal amounts of protein were used for Western blot analysis using either an anti-HA, anti-myc or anti-tubulin antibody (lower three panels). Lysates were also subjected to immunoprecipitation (IP) analysis using an immobilized anti-myc antibody and Fn14-HA:Fn14-myc association analyzed by Western blot analysis with an anti-HA antibody (top two panels). The positions of molecular size markers are shown on the left (in kDa).

It has been reported that when CD40 [54] and CD30 [52] deletion mutants lacking TRAF binding sites in the cytoplasmic domain are overexpressed in cells they can act as dominant-negative inhibitors of ligand-stimulated signaling, presumably by associating with endogenous receptors and thereby forming non-functional receptor complexes. In a previous study, we reported that MDA-MB-231 breast cancer cell invasion capacity could be inhibited by forced Fn14-ΔCT expression [32], suggesting that Fn14-ΔCT may also be a dominant-negative inhibitor. Therefore, we wanted to determine whether Fn14-ΔCT could associate with the Fn14-FL protein even though it cannot associate with itself. For these studies, we used a co-immunoprecipitation approach. HEK293 cells engineered to stably overexpress HA epitope-tagged Fn14-FL (Fig. 5A) were transfected with vector or the Fn14-FL, Fn14-ΔEC, or Fn14-ΔCT expression plasmids (all proteins myc-tagged) and 24 hr later the cells were harvested. Western blot analysis was performed on an aliquot of each lysate using either an anti-HA or anti-myc antibody to confirm expression of all Fn14 proteins (Fig. 5B). Lysates were also subjected to immunoprecipitation analysis using an immobilized anti-myc antibody and Fn14-HA:Fn14-myc association evaluated by Western blot analysis with an anti-HA antibody. We found that all three myc-tagged Fn14 proteins were effectively immunoprecipitated with the anti-myc antibody and could interact with HA-tagged Fn14-FL (Fig. 5B).

These results indicate that the effect of Fn14-ΔCT in our earlier study [32] most likely reflects Fn14-ΔCT association with endogenous Fn14, leading to the formation of signaling-deficient heteromeric complexes. Indeed, one would predict that ectopic Fn14-ΔCT expression in cells would interfere with both TWEAK-dependent and -independent Fn14 signaling. Our finding that Fn14-ΔEC can also associate with Fn14-FL suggests that if this Fn14 splice variant isoform is naturally co-expressed at significant levels in cells with the Fn14-FL protein, it could act as a dominant-negative inhibitor of TWEAK binding to Fn14 (i.e., TWEAK-dependent Fn14 signaling). However, since Fn14-ΔEC can signal on its own when overexpressed (Fig. 3C), one would not expect it to interfere with TWEAK-independent Fn14 signaling.

### Fn14 Dimers are Detected when Cell Lysates are Analyzed by SDS-PAGE Under Non-reducing Conditions

**Figure 6:**
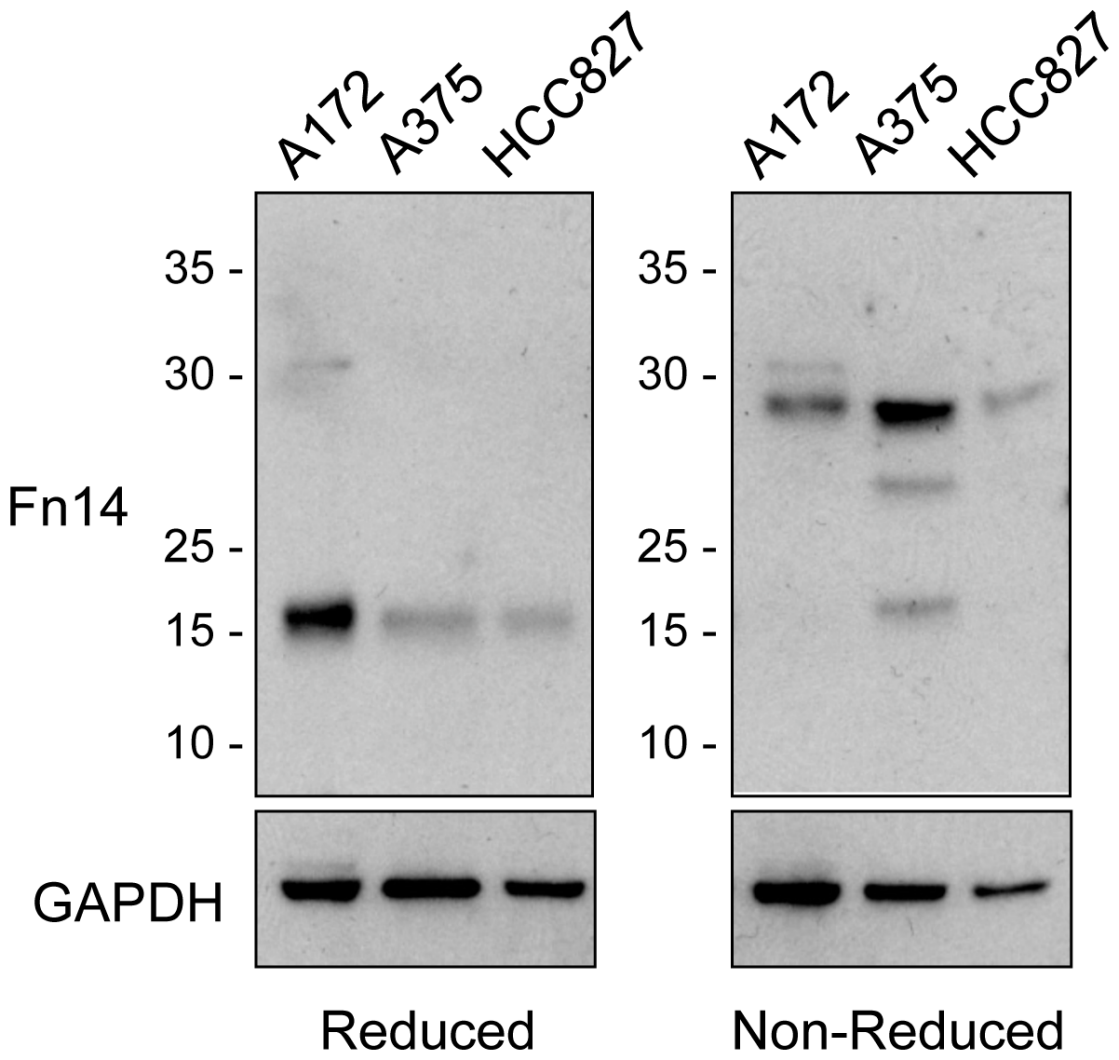
Endogenously expressed Fn14 has slower electrophoretic mobility when analyzed under non-reducing conditions. Cell lysates were prepared from the indicated cell lines and equal amounts of protein were subjected to SDS-PAGE under either reducing or non-reducing conditions. Western blot analysis was performed using either an anti-Fn14 antibody (from CST) or an anti-GAPDH antibody. The positions of molecular size markers are shown on the left (in kDa).

We noted that in our ligand blot experiments, in which cell lysate samples are run under non-reducing conditions (no DTT in loading buffer, sample is not heated), the overexpressed Fn14-FL protein had slower electrophoretic mobility when compared to its mobility under normal (reducing) SDS-PAGE conditions (Fig. 2B). Therefore, we examined whether endogenously-expressed Fn14 also had different electrophoretic mobility under these two conditions. Lysates were prepared from three different cancer cell lines, protein was subjected to SDS-PAGE under either reducing or non-reducing conditions, and Western blot analysis was performed. In all three cell lines, Fn14 migrated as a single species with an apparent molecular mass of ∼17-kDa under reducing conditions (Fig. 6). In comparison, under non-reducing conditions, the major immunoreactive Fn14 species detected in the cell lines was ∼29-kDa, with two lower molecular mass species of ∼26- and ∼17-kDa also present in the A375 cell lysate. These results indicate that endogenous Fn14 could be forming a disulfide-linked dimer, either in the cell itself or after cell harvest during the cell lysis procedure.

### Fn14 Dimerization Occurs during Cell Lysis via Formation of an Intermolecular Disulfide Bond at Cysteine Residue 122

**Figure 7:**
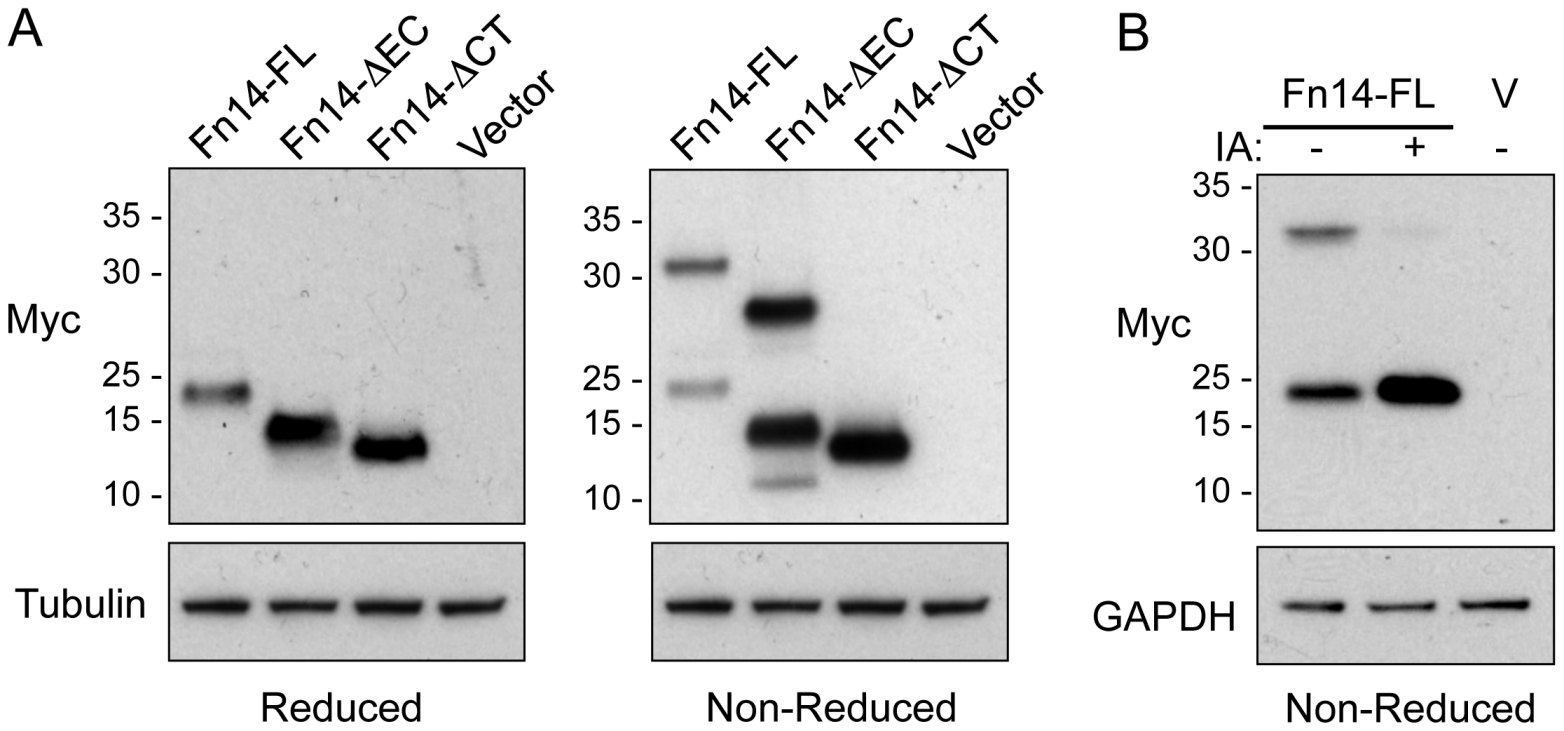
The Fn14-FL and Fn14-ΔEC proteins, but not the Fn14-ΔCT protein, have slower electrophoretic mobility when analyzed under non-reducing conditions due to intermolecular disulfide bond formation during cell lysis. (**A**) HEK293 cells were transfected with vector or the indicated Fn14-myc expression plasmids and 24 hr later the cells were harvested, lysed, and equal amounts of protein were subjected to SDS-PAGE under either reducing or non-reducing conditions. Western blot analysis was performed using either an anti-myc or anti-tubulin antibody. The positions of molecular size markers are shown on the left (in kDa). (**B**) HEK293 cells were transfected with vector (V) or the Fn14-FL-myc expression plasmid and 24 hr later the cells were harvested. Cells were lysed and an aliquot of the Fn14-FL lysate was incubated with iodoacetamide (IA). Equal amounts of protein were subjected to SDS-PAGE under non-reducing conditions and Western blot analysis was performed using either an anti-myc or anti-GAPDH antibody. The positions of molecular size markers are shown on the left (in kDa).

Fn14 contains a total of eight cysteine residues. Six of these residues are in the extracellular region, where they form the three intramolecular disulfide bonds that stabilize the cysteine-rich domain [41]–[43]. The other two cysteines, Cys104 and Cys122, are located in the cytoplasmic tail, and since disulfide bonds are formed in the lumen of the rough ER during protein synthesis, they are not expected to form intra- or intermolecular disulfide bonds in living cells. In order to determine which Fn14 cysteine residues were likely involved in the endogenous Fn14 dimerization noted above, HEK293 cells were transfected with vector or the Fn14-FL (contains all 8 cysteines), Fn14-ΔEC (contains only the 2 cysteines in the cytoplasmic tail), or Fn14-ΔCT (contains all 6 cysteines in the extracellular domain and Cys104 in the cytoplasmic tail) plasmids and 24 hr later the cells were harvested and lysed. Samples were subjected to SDS-PAGE under either reducing or non-reducing conditions and Western blot analysis was performed. Fn14-FL and Fn14-ΔEC dimers, but not Fn14-ΔCT dimers, were detected under non-reducing conditions (Fig. 7A). This result indicated that the most C-terminal Fn14 cysteine residue, Cys122, was critical for disulfide bond formation. Since this residue is within the cytoplasmic domain, this result also implied that disulfide bond formation was occurring after cell harvest. To confirm this, HEK293 cells were transfected with vector or the Fn14-FL protein and 24 hr later the cells were harvested. The Fn14-FL cells were then lysed in normal lysis buffer or lysis buffer supplemented with iodoacetamide, which reacts with free sulfhydryls on cysteine residues. Samples were subjected to SDS-PAGE under non-reducing conditions, and Western blot analysis was performed. Iodoacetamide treatment blocked Fn14-FL dimer formation (Fig. 7B), indicating that the Cys122 intermolecular disulfide bond was forming in the cell lysis buffer, a favorable redox environment for oxidation of SH groups.

**Figure 8:**
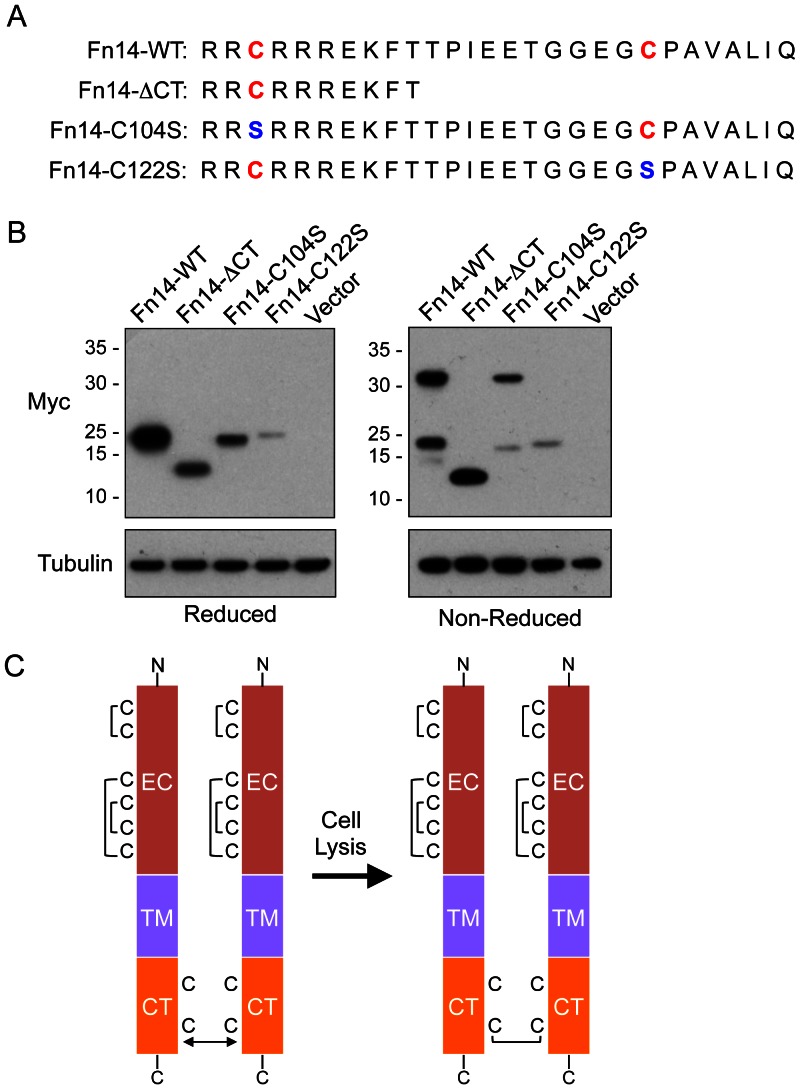
Fn14-FL dimerization during cell lysis can be abrogated by mutagenesis of cysteine residue 122. (**A**) The human Fn14-WT (FL), Fn14-ΔCT, Fn14-C104S, and Fn14-C122S cytoplasmic tail amino acid sequences are shown with cysteine residues in red and the serine residue substitutions in blue. (**B**) HEK293 cells were transfected with vector or the indicated Fn14-myc expression plasmids and 24 hr later the cells were harvested and lysed. Equal amounts of protein were subjected to SDS-PAGE under either reducing or non-reducing conditions and Western blot analysis was performed using either an anti-myc or anti-tubulin antibody. The positions of molecular size markers are shown on the left (in kDa). (**C**) Schematic representation of predicted Fn14 cysteine residue status prior to and after cell harvesting and lysis. The Fn14 extracellular (EC) domain, transmembrane (TM) domain, and cytoplasmic tail (CT) are indicated and the cysteine residue distribution within the Fn14-FL protein is shown. Disulfide bonds are indicated with brackets. Non-covalent Fn14 association mediated by the most distal 18-aa residues of the Fn14 CT is illustrated with a double-headed arrow.

We next confirmed that Cys122 within the Fn14 cytoplasmic domain was indeed the residue participating in intermolecular disulfide bond formation by site-specific mutagenesis. Expression plasmids were constructed that encoded myc-tagged Fn14 proteins with a cysteine to serine substitution at position 104 or 122 (Fig. 8A). HEK293 cells were transiently transfected with each of these plasmids, as well as the Fn14-FL (denoted wild-type (WT) in this figure) and Fn14-ΔCT plasmids used earlier. Cells were harvested, lysed, and samples were subjected to SDS-PAGE under either reducing or non-reducing conditions. Western blot analysis was performed. All four of the Fn14 proteins were expressed in the transfected cells and were detected as single species when analyzed under reducing conditions (Fig. 8B). Fn14-WT and Fn14-C104S dimers, but not Fn14-ΔCT or Fn14-C122S dimers, were detected under non-reducing conditions, confirming the importance of the C-terminal region of the cytoplasmic tail and Cys122 in particular in intermolecular disulfide bond formation (Fig. 8B).

### Conclusions

In this study, we first show that there are at least two distinct Fn14 mRNA species, with one mRNA encoding the full-length 129-aa Fn14 protein and the second mRNA, generated by alternative splicing of the primary transcript, encoding a smaller 94-aa Fn14 protein missing most of the extracellular domain and therefore unable to bind the Fn14 ligand TWEAK. The cloning of the smaller protein, denoted Fn14-ΔEC, afforded us the opportunity to test our earlier proposal that high levels of Fn14 expression; for example, in mouse intestinal epithelial cells after tissue injury [19] or in human non-small cell lung cancer cells with EGFR-activating mutations [24], might trigger TWEAK-independent Fn14 signaling. We found that ectopic overexpression of Fn14-ΔEC could indeed activate the NF-κB signaling pathway. We then demonstrated that ectopic overexpression of Fn14 promoted self-association on the cell surface using a chemical cross-linking approach. Specifically, cell surface Fn14 was in a dimeric structure that was stabilized by the most distal 18-aa region of the Fn14 cytoplasmic tail. Endogenously-expressed Fn14 as well as ectopically-overexpressed Fn14 could also be detected in dimeric form when cell lysates were subjected to SDS-PAGE under non-reducing conditions. Additional experiments revealed that Fn14 dimerization occurs during cell lysis via formation of an intermolecular disulfide bond at cysteine residue 122. Taken together, our results support the model that both endogenous Fn14 and ectopically-overexpressed Fn14 monomers can self-assemble within cells into non-covalently associated dimers (Fig. 8C). These Fn14 monomers remain in such close proximity in cell lysis buffer that a disulfide bond linkage is able to form at Fn14 cysteine residue 122 in the cytoplasmic tail. These findings provide new insights into the TWEAK/Fn14 signaling pathway and will aid in the design, development, and testing of therapeutic agents targeting this pathway.

## References

[1] Meighan-Mantha RL, Hsu DKW, Guo Y, Brown SAN, Feng SY, et al (1999) The mitogen-inducible Fn14 gene encodes a type I transmembrane protein that modulates fibroblast adhesion and migration. J Biol Chem 274: 33166–33176. 10551889 10.1074/jbc.274.46.33166

[2] Feng SY, Guo Y, Factor VM, Thorgeirsson SS, Bell DW, et al (2000) The Fn14 immediate-early response gene is induced during liver regeneration and highly expressed in both human and murine hepatocellular carcinomas. Am J Pathol 156: 1253–1261. 10751351 10.1016/S0002-9440(10)64996-6 PMC1876890

[3] Wiley SR, Cassiano L, Lofton T, Davis-Smith T, Winkles JA, et al (2001) A novel TNF receptor family member binds TWEAK and is implicated in angiogenesis. Immunity 15: 837–846. 11728344 10.1016/s1074-7613(01)00232-1

[4] Winkles JA (2008) The TWEAK-Fn14 cytokine-receptor axis: Discovery, biology and therapeutic targeting. Nat Rev Drug Discov 7: 411–425. 18404150 10.1038/nrd2488 PMC3018765

[5] Burkly LC, Michaelson JS, Zheng TS (2011) TWEAK/Fn14 pathway: An immunological switch for shaping tissue responses. Immunol Rev 244: 99–114. 22017434 10.1111/j.1600-065X.2011.01054.x

[6] Bossen C, Ingold K, Tardivel A, Bodmer JL, Gaide O, et al (2006) Interactions of tumor necrosis factor (TNF) and TNF receptor family members in the mouse and human. J Biol Chem 281: 13964–13971. 16547002 10.1074/jbc.M601553200

[7] Varfolomeev E, Goncharov T, Maecker H, Zobel K, Komuves LG, et al (2012) Cellular inhibitors of apoptosis are global regulators of NF-κB and MAPK activation by members of the TNF family of receptors. Sci Signal 5: ra22. 22434933 10.1126/scisignal.2001878

[8] Donohue PJ, Richards CM, Brown SA, Hanscom HN, Buschman J, et al (2003) TWEAK is an endothelial cell growth and chemotactic factor that also potentiates FGF-2 and VEGF-A mitogenic activity. Arterioscler Thromb Vasc Biol 23: 594–600. 12615668 10.1161/01.ATV.0000062883.93715.37

[9] Li H, Mittal A, Paul PK, Kumar M, Srivastava DS, et al (2009) Tumor necrosis factor-related weak inducer of apoptosis augments matrix metalloproteinase 9 (MMP-9) production in skeletal muscle through the activation of nuclear factor-κB-inducing kinase and p38 mitogen-activated protein kinase: A potential role of MMP-9 in myopathy. J Biol Chem 284: 4439–4450. 19074147 10.1074/jbc.M805546200 PMC2640955

[10] Kumar M, Makonchuk DY, Li H, Mittal A, Kumar A (2009) TNF-like weak inducer of apoptosis (TWEAK) activates proinflammatory signaling pathways and gene expression through the activation of TGF-β-activated kinase 1. J Immunol 182: 2439–2448. 19201899 10.4049/jimmunol.0803357 PMC2652039

[11] Fortin SP, Ennis MJ, Savitch BA, Carpentieri D, McDonough WS, et al (2009) Tumor necrosis factor-like weak inducer of apoptosis stimulation of glioma cell survival is dependent on Akt2 function. Mol Cancer Res 7: 1871–1881. 19861406 10.1158/1541-7786.MCR-09-0194 PMC2783270

[12] Tran NL, McDonough WS, Savitch BA, Fortin SP, Winkles JA, et al (2006) Increased fibroblast growth factor-inducible 14 expression levels promote glioma cell invasion via Rac1 and nuclear factor-κB and correlate with poor patient outcome. Cancer Res 66: 9535–9542. 17018610 10.1158/0008-5472.CAN-06-0418

[13] Saitoh T, Nakayama M, Nakano H, Yagita H, Yamamoto N, et al (2003) TWEAK induces NF-κB2 p100 processing and long lasting NF-κB activation. J Biol Chem 278: 36005–36012. 12840022 10.1074/jbc.M304266200

[14] Vince JE, Chau D, Callus B, Wong WW, Hawkins CJ, et al (2008) TWEAK-Fn14 signaling induces lysosomal degradation of a cIAP1-TRAF2 complex to sensitize tumor cells to TNF-α. J Cell Biol 182: 171–184. 18606850 10.1083/jcb.200801010 PMC2447903

[15] Sanz AB, Sanchez-Nino MD, Izquierdo MC, Jakubowski A, Justo P, et al (2010) TWEAK activates the non-canonical NF-κB pathway in murine renal tubular cells: Modulation of CCL21. PLoS One 5: e8955. 20126461 10.1371/journal.pone.0008955 PMC2813291

[16] Brown SA, Ghosh A, Winkles JA (2010) Full-length, membrane-anchored TWEAK can function as a juxtacrine signaling molecule and activate the NF-κB pathway. J Biol Chem 285: 17432–17441. 20385556 10.1074/jbc.M110.131979 PMC2878507

[17] Maecker H, Varfolomeev E, Kischkel F, Lawrence D, LeBlanc H, et al (2005) TWEAK attenuates the transition from innate to adaptive immunity. Cell 123: 931–944. 16325585 10.1016/j.cell.2005.09.022

[18] Jakubowski A, Ambrose C, Parr M, Lincecum JM, Wang MZ, et al (2005) TWEAK induces liver progenitor cell proliferation. J Clin Invest 115: 2330–2340. 16110324 10.1172/JCI23486 PMC1187931

[19] Dohi T, Borodovsky A, Wu P, Shearstone JR, Kawashima R, et al (2009) TWEAK/Fn14 pathway: A nonredundant role in intestinal damage in mice through a TWEAK/intestinal epithelial cell axis. Gastroenterology 136: 912–923. 19109961 10.1053/j.gastro.2008.11.017

[20] Winkles JA, Tran NL, Brown SA, Stains N, Cunliffe HE, et al (2007) Role of TWEAK and Fn14 in tumor biology. Front Biosci 12: 2761–2771. 17127278 10.2741/2270

[21] Winkles JA, Tran NL, Berens ME (2005) TWEAK and Fn14: New molecular targets for cancer therapy? Cancer Lett 235: 11–17. 10.1016/j.canlet.2005.03.048 15885893

[22] Burkly LC, Michaelson JS, Hahm K, Jakubowski A, Zheng TS (2007) TWEAKing tissue remodeling by a multifunctional cytokine: Role of TWEAK/Fn14 pathway in health and disease. Cytokine 40: 1–16. 17981048 10.1016/j.cyto.2007.09.007

[23] Culp PA, Choi D, Zhang Y, Yin J, Seto P, et al (2010) Antibodies to TWEAK receptor inhibit human tumor growth through dual mechanisms. Clin Cancer Res 16: 497–508. 20068083 10.1158/1078-0432.CCR-09-1929

[24] Whitsett TG, Cheng E, Inge L, Asrani K, Jameson NM, et al (2012) Elevated expression of Fn14 in non-small cell lung cancer correlates with activated EGFR and promotes tumor cell migration and invasion. Am J Pathol 181: 111–120. 22634180 10.1016/j.ajpath.2012.03.026 PMC3388162

[25] Zhou H, Ekmekcioglu S, Marks JW, Mohamedali KA, Asrani K, et al (2013) The TWEAK receptor Fn14 is a therapeutic target in melanoma: Immunotoxins targeting Fn14 receptor for malignant melanoma treatment. J Invest Dermatol 133: 1052–1062. 23190886 10.1038/jid.2012.402 PMC3600159

[26] Chao DT, Su M, Tanlimco S, Sho M, Choi D, et al (2013) Expression of TweakR in breast cancer and preclinical activity of enavatuzumab, a humanized anti-TweakR mAb. J Cancer Res Clin Oncol 139: 315–325. 23073510 10.1007/s00432-012-1332-x PMC3549414

[27] Michaelson JS, Amatucci A, Kelly R, Su L, Garber E, et al (2011) Development of an Fn14 agonistic antibody as an anti-tumor agent. MAbs 3: 362–375. 21697654 10.4161/mabs.3.4.16090 PMC3218533

[28] Michaelson JS, Kelly R, Yang L, Zhang X, Wortham K, et al (2012) The anti-Fn14 antibody BIIB036 inhibits tumor growth in xenografts and patient derived primary tumor models and enhances efficacy of chemotherapeutic agents in multiple xenograft models. Cancer Biol Ther 13: 812–821. 22669574 10.4161/cbt.20564

[29] Ho DH, Vu H, Brown SAN, Donohue PJ, Hanscom HN, et al (2004) Soluble tumor necrosis factor-like weak inducer of apoptosis overexpression in HEK293 cells promotes tumor growth and angiogenesis in athymic nude mice. Cancer Res 64: 8968–8972. 15604260 10.1158/0008-5472.CAN-04-1879

[30] Zhou H, Marks JW, Hittelman WN, Yagita H, Cheung LH, et al (2011) Development and characterization of a potent immunoconjugate targeting the Fn14 receptor on solid tumor cells. Mol Cancer Ther 10: 1276–1288. 21586630 10.1158/1535-7163.MCT-11-0161 PMC3523329

[31] Tran NL, McDonough WS, Donohue PJ, Winkles JA, Berens TJ, et al (2003) The human Fn14 receptor gene is up-regulated in migrating glioma cells *in vitro* and overexpressed in advanced glial tumors. Am J Pathol 162: 1313–1321. 12651623 10.1016/S0002-9440(10)63927-2 PMC1851233

[32] Willis AL, Tran NL, Chatigny JM, Charlton N, Vu H, et al (2008) The fibroblast growth factor-inducible 14 receptor is highly expressed in HER2-positive breast tumors and regulates breast cancer cell invasive capacity. Mol Cancer Res 6: 725–734. 18505918 10.1158/1541-7786.MCR-08-0005 PMC3519279

[33] Tran NL, McDonough WS, Savitch BA, Sawyer TF, Winkles JA, et al (2005) The tumor necrosis factor-like weak inducer of apoptosis (TWEAK)-fibroblast growth factor-inducible 14 (Fn14) signaling system regulates glioma cell survival via NF-κB pathway activation and BCL-XL/BCL-W expression. J Biol Chem 280: 3483–3492. 15611130 10.1074/jbc.M409906200

[34] Watts GS, Tran NL, Berens ME, Bhattacharyya AK, Nelson MA, et al (2007) Identification of Fn14/TWEAK receptor as a potential therapeutic target in esophageal adenocarcinoma. Int J Cancer 121: 2132–2139. 17594693 10.1002/ijc.22898

[35] Huang M, Narita S, Tsuchiya N, Ma Z, Numakura K, et al (2011) Overexpression of Fn14 promotes androgen-independent prostate cancer progression through MMP-9 and correlates with poor treatment outcome. Carcinogenesis 32: 1589–1596. 21828059 10.1093/carcin/bgr182

[36] Fortin SP, Ennis MJ, Schumacher CA, Zylstra-Diegel CR, Williams BO, et al (2012) Cdc42 and the guanine nucleotide exchange factors Ect2 and Trio mediate Fn14-induced migration and invasion of glioblastoma cells. Mol Cancer Res 10: 958–968. 22571869 10.1158/1541-7786.MCR-11-0616 PMC3516844

[37] Blanco-Colio LM, Martin-Ventura JL, Munoz-Garcia B, Orbe J, Paramo JA, et al (2007) Identification of soluble tumor necrosis factor-like weak inducer of apoptosis (sTWEAK) as a possible biomarker of subclinical atherosclerosis. Arterioscler Thromb Vasc Biol 27: 916–922. 17272745 10.1161/01.ATV.0000258972.10109.ff

[38] Jain M, Jakubowski A, Cui L, Shi J, Su L, et al (2009) A novel role for tumor necrosis factor-like weak inducer of apoptosis (TWEAK) in the development of cardiac dysfunction and failure. Circulation 119: 2058–2068. 19349318 10.1161/CIRCULATIONAHA.108.837286 PMC2924152

[39] Rubin JB, Kung AL, Klein RS, Chan JA, Sun Y, et al (2003) A small-molecule antagonist of CXCR4 inhibits intracranial growth of primary brain tumors. Proc Nat Acad Sci USA 100: 13513–13518. 14595012 10.1073/pnas.2235846100 PMC263845

[40] Keren H, Lev-Maor G, Ast G (2010) Alternative splicing and evolution: Diversification, exon definition and function. Nat Rev Genet 11: 345–355. 20376054 10.1038/nrg2776

[41] Brown SAN, Hanscom HN, Vu H, Brew SA, Winkles JA (2006) TWEAK binding to the Fn14 cysteine-rich domain depends on charged residues located in both the A1 and D2 modules. Biochem J 397: 297–304. 16526941 10.1042/BJ20051362 PMC1513280

[42] Foley SF, Sun Y, Zheng TS, Wen D (2008) Picomole-level mapping of protein disulfides by mass spectrometry following partial reduction and alkylation. Anal Biochem 377: 95–104. 18358819 10.1016/j.ab.2008.02.025

[43] He F, Dang W, Saito K, Watanabe S, Kobayashi N, et al (2009) Solution structure of the cysteine-rich domain in Fn14, a member of the tumor necrosis factor receptor superfamily. Protein Sci 18: 650–656. 19241374 10.1002/pro.49 PMC2760370

[44] Brown SAN, Richards CM, Hanscom HN, Feng SL, Winkles JA (2003) The Fn14 cytoplasmic tail binds tumour necrosis factor receptor associated factors 1, 2, 3 and 5 and mediates nuclear factor-κB activation. Biochem J 371: 395–403. 12529173 10.1042/BJ20021730 PMC1223299

[45] Han S, Yoon K, Lee K, Kim K, Jang H, et al (2003) TNF-related weak inducer of apoptosis receptor, a TNF receptor superfamily member, activates NF-κB through TNF receptor-associated factors. Biochem Biophys Res Commun 305: 789–796. 12767899 10.1016/s0006-291x(03)00852-0

[46] Locksley RM, Killeen N, Lenardo MJ (2001) The TNF and TNF receptor superfamilies: Integrating mammalian biology. Cell 104: 487–501. 11239407 10.1016/s0092-8674(01)00237-9

[47] Valley CC, Lewis AK, Mudaliar DJ, Perlmutter JD, Braun AR, et al (2012) Tumor necrosis factor-related apoptosis-inducing ligand (TRAIL) induces death receptor 5 networks that are highly organized. J Biol Chem 287: 21265–21278. 22496450 10.1074/jbc.M111.306480 PMC3375548

[48] Chan FK, Chun HJ, Zheng L, Siegel RM, Bui KL, et al (2000) A domain in TNF receptors that mediates ligand-independent receptor assembly and signaling. Science 288: 2351–2354. 10875917 10.1126/science.288.5475.2351

[49] Yousaf N, Gould DJ, Aganna E, Hammond L, Mirakian RM, et al (2005) Tumor necrosis factor receptor I from patients with tumor necrosis factor receptor-associated periodic syndrome interacts with wild-type tumor necrosis factor receptor I and induces ligand-independent NF-κB activation. Arthritis Rheum 52: 2906–2916. 16142754 10.1002/art.21268

[50] Chan FK (2007) Three is better than one: Pre-ligand receptor assembly in the regulation of TNF receptor signaling. Cytokine 37: 101–107. 17449269 10.1016/j.cyto.2007.03.005 PMC1965282

[51] Naismith JH, Devine TQ, Brandhuber BJ, Sprang SR (1995) Crystallographic evidence for dimerization of unliganded tumor necrosis factor receptor. J Biol Chem 270: 13303–13307. 7768931 10.1074/jbc.270.22.13303

[52] Horie R, Watanabe T, Morishita Y, Ito K, Ishida T, et al (2002) Ligand-independent signaling by overexpressed CD30 drives NF-κB activation in Hodgkin-Reed-Sternberg cells. Oncogene 21: 2493–2503. 11971184 10.1038/sj.onc.1205337

[53] Kanazawa K, Kudo A (2005) Self-assembled RANK induces osteoclastogenesis ligand-independently. J Bone Miner Res 20: 2053–2060. 16234979 10.1359/JBMR.050706

[54] Mehl AM, Jones M, Rowe M, Brennan P (2001) Characterization of a CD40-dominant inhibitory receptor mutant. J Immunol 167: 6388–6393. 11714804 10.4049/jimmunol.167.11.6388

